# Hypertension management in rural western Kenya: a needs-based health workforce estimation model

**DOI:** 10.1186/s12960-019-0389-x

**Published:** 2019-07-16

**Authors:** Rajesh Vedanthan, Danielle J. Lee, Jemima H. Kamano, Omarys I. Herasme, Peninah Kiptoo, Deborah Tulienge, Sylvester Kimaiyo, Hari Balasubramanian, Valentin Fuster

**Affiliations:** 1 New York University School of Medicine, 180 Madison Avenue, 8th Floor, New York, NY 10016 USA; 2 Temple University School of Medicine, 3500 N Broad St, Philadelphia, PA 19140 USA; 3 Department of Medicine, School of Medicine, Moi University College of Health Sciences, Nandi Rd, Eldoret, Kenya; 4 Icahn School of Medicine at Weill Cornell, 1 Gustave L. Levy Pl, New York, NY 10029 USA; 5 Academic Model Providing Access to Healthcare (AMPATH), P.O. Box 4606, Eldoret, 30100 Kenya; 6 University of Massachusetts, 306 Godell, Amherst, MA 01003 USA; 7 Weill Cornell Medicine, 413 East 69th Street, New York, NY 10021 USA

**Keywords:** Hypertension, Low- and middle-income countries, Workforce estimation, Task redistribution

## Abstract

**Background:**

Elevated blood pressure is the leading risk for mortality in the world. Task redistribution has been shown to be efficacious for hypertension management in low- and middle-income countries. However, the workforce requirements for such a task redistribution strategy are largely unknown. Therefore, we developed a needs-based workforce estimation model for hypertension management in western Kenya, using need and capacity as inputs.

**Methods:**

Key informant interviews, focus group discussions, a Delphi exercise, and time-motion studies were conducted among administrative leadership, clinicians, patients, community leaders, and experts in hypertension management. These results were triangulated to generate the best estimates for the inputs into the health workforce model. The local hypertension clinical protocol was used to derive a schedule of encounters with different levels of clinician and health facility staff. A Microsoft Excel-based spreadsheet was developed to enter the inputs and generate the full-time equivalent workforce requirement estimates over 3 years.

**Results:**

Two different scenarios were modeled: (1) “ramp-up” (increasing growth of patients each year) and (2) “steady state” (constant rate of patient enrollment each month). The ramp-up scenario estimated cumulative enrollment of 7000 patients by year 3, and an average clinical encounter time of 8.9 min, yielding nurse full-time equivalent requirements of 4.8, 13.5, and 30.2 in years 1, 2, and 3, respectively. In contrast, the steady-state scenario assumed a constant monthly enrollment of 100 patients and yielded nurse full-time equivalent requirements of 5.8, 10.5, and 14.3 over the same time period.

**Conclusions:**

A needs-based workforce estimation model yielded health worker full-time equivalent estimates required for hypertension management in western Kenya. The model is able to provide workforce projections that are useful for program planning, human resource allocation, and policy formulation. This approach can serve as a benchmark for chronic disease management programs in low-resource settings worldwide.

**Electronic supplementary material:**

The online version of this article (10.1186/s12960-019-0389-x) contains supplementary material, which is available to authorized users.

## Background

The global burden of hypertension predominates in low- and middle-income countries (LMICs), with 80% of hypertension-related deaths occurring in those regions [1]. However, treatment and control rates in these regions are poor [2, 3], due to inequitable access to health care, failure to uptitrate anti-hypertensive therapy, co-morbidities (e.g., obesity and diabetes), insufficient lifestyle modification, poor medication adherence, and psychosocial factors [4–9].

In addition to these barriers, LMICs also have insufficient human resources for health [10]. Given the severe shortage of physicians in LMICs, task redistribution of hypertension care from physicians to non-physician health workers could improve hypertension treatment and control rates in low-resource settings worldwide [11, 12]. However, the workforce requirements for such a task redistribution strategy are largely unknown.

In this paper, we describe the development of a needs-based workforce estimation model for hypertension management in Kenya, modeled after a similar workforce estimation model developed for human immunodeficiency virus (HIV) care in Mozambique [13]. We aimed to estimate the number of physicians, nurses, clinical officers, and auxiliary personnel necessary to provide stable, long-term care for patients with hypertension in this resource-limited setting.

## Methods

### Aim

To develop a needs-based workforce estimation model for hypertension management in western Kenya, using need and capacity as inputs.

### Setting

Since its initiation in Kenya in 2001, the Academic Model Providing Access to Healthcare Partnership (AMPATH) has established an HIV care system that has served over 160,000 patients [14]. Recently, in collaboration with the government of Kenya, AMPATH has broadened its clinical scope of work to include hypertension care [15, 16]. Our research study was conducted within the AMPATH catchment area in the Kosirai and Turbo divisions of western Kenya. Each division has one rural health center and several more decentralized rural dispensaries.

### Conceptual model

We developed a needs-based workforce estimation model that took into account need and capacity, resulting in full-time equivalent health workers. The definition and formula for each of these are as follows:

1. Need: If *i* denotes patient category and *j* denotes type of provider, then *need* is the total number of clinical encounters per month for provider *j* (*v*_*j*_) required to care for all hypertension patients in category *i* (*n*_*ij*_), depending on the different encounter frequency (*f*_*ij*_) that patient category *i* needs with provider type *j*.

1 $$ {v}_j={\Sigma}_i\ \left({f}_{ij}\times {n}_{ij}\right) $$

2. *Capacity* is the number of patient encounters possible per month (*E*_*j*_) for a provider, depending on the amount of patient-contact time (*t*_*j*_) each type of provider can work per month and the average productivity of each type of provider (time per clinical encounter (*m*_*j*_)).

2 $$ \mathrm{Capacity}:{E}_j={t}_j/{m}_j $$

3. The final output is *full-time equivalents* (FTEs) (*F*_*j*_) required for a type of provider to meet the total need:

3 $$ {F}_j={v}_j/{E}_j $$

### Data collection

**Fig. 1:**
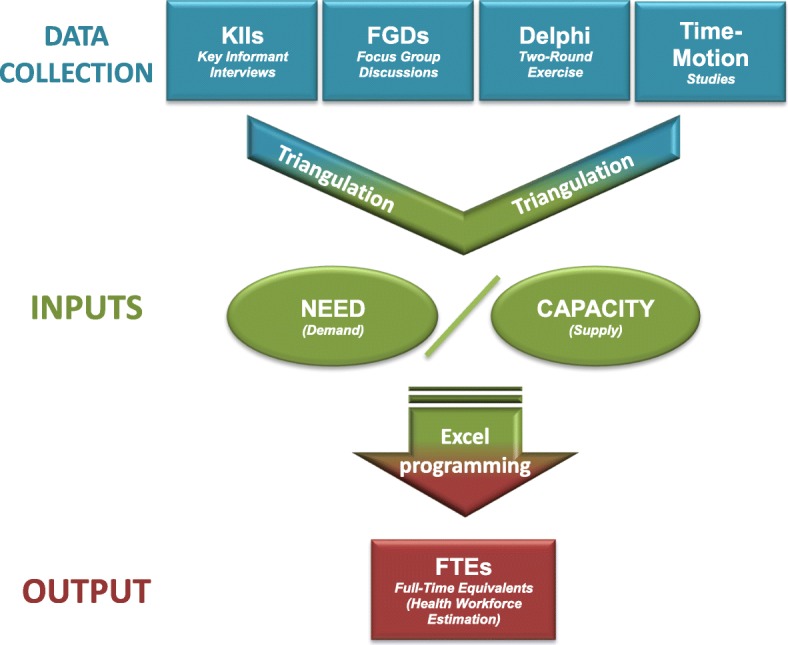
Overall approach to the study, illustrating the data collection elements, triangulation to create the model inputs, and generation of the model output using the Microsoft Excel-based worksheet.

A combination of key informant interviews, focus group discussions, a Delphi exercise, and time-motion studies were utilized to generate the data required to calculate the inputs into the health workforce model (Fig. 1). Results from each of these techniques were triangulated to generate the best estimates for inputs into the model.

Key informant interviews: Key informant interviews (KIIs) were conducted with AMPATH’s Chronic Disease Management (CDM) leadership, patients, and community leaders (*N* = 7). The interviews took place at a private location convenient for the interviewees. Prior to initiating the KIIs, verbal informed consent was obtained from each study participant. All interviews were audio-recorded verbatim, with the research staff member taking notes.

Focus group discussions: Focus group discussions (FGDs) were conducted with health workers and patients (*N* = 6). The FGDs were scheduled at a location that was mutually convenient for all participants. Verbal informed consent was obtained from each FGD study participant. All group discussions were audio-recorded verbatim, and the moderator took notes during the discussions.

Both the semi-structured KIIs and the FGDs aimed at exploring the participants’ assessments of the type and number of personnel that would be required for a nurse management of hypertension program at that point in time, as well as projected into the next several years. The audio-recordings from all of the sessions were transcribed and translated into English by an interpreter. Content analysis of the transcribed interviews, discussions, and moderator notes was performed by first assigning unique codes based on content [17], according to the following deductive (a priori) codes: (1) estimated and anticipated number of hypertension patients to be managed, (2) estimated and anticipated number of nurses required to provide hypertension care; (3) types and number of other personnel required for hypertension management, and (4) workflow and work-time allocation. Significant inductive (emerging) codes were also identified. Codes were subsequently grouped together by theme, and the relationships between and among them were formulated.

Delphi exercise: We conducted a two-round Delphi exercise involving eight invited experts in hypertension management, health care in Kenya, or behavioral research. The Delphi exercise is a consensus-building, group facilitation technique that collects expert opinions through several rounds of surveys or interviews and is characterized by anonymity, iteration, controlled feedback, and statistical group response (expression of the degree of consensus within a group) [18, 19]. Exemption from informed consent among this study group was requested and approved due to the minimal risk and absence of any personal health information involved with this technique.

In the first round, each participant was asked to estimate the number and complexity of hypertension patients in western Kenya, health workforce requirements to manage hypertension, and expected 1-year outcome for such patients. In the second round, each participant has presented the first-round responses of all other participants in an anonymous fashion and again asked to answer the same questions. At the conclusion of the second round, the estimates for each of the items were summarized using descriptive statistics (mean and range).

Time-motion studies: Time-motion studies were conducted in order to supplement information relating to capacity. Patients seen at rural dispensaries within the AMPATH catchment area of Kosirai and Turbo were eligible to participate in this component of the study. After written informed consent was obtained from each participant, a research staff member timed the duration of a clinical encounter between each patient and the rural clinician. The research team member did not enter the examination room during the health worker’s interaction with the patient, but rather observed and took notes from outside the room at the start and end of the clinical encounter. The *Time-Motion Study* application by Graphite, Inc. (Sacramento, CA), was used to record the time of each clinical encounter. One-hundred-and-eighteen clinical encounters were observed over seven days in seven rural dispensaries—66 patients with hypertension and 52 patients without hypertension. Each patient’s demographic and clinical information was also extracted from the clinical record in an anonymous fashion. The results were summarized using descriptive statistics.

### Model development

**Fig. 2:**
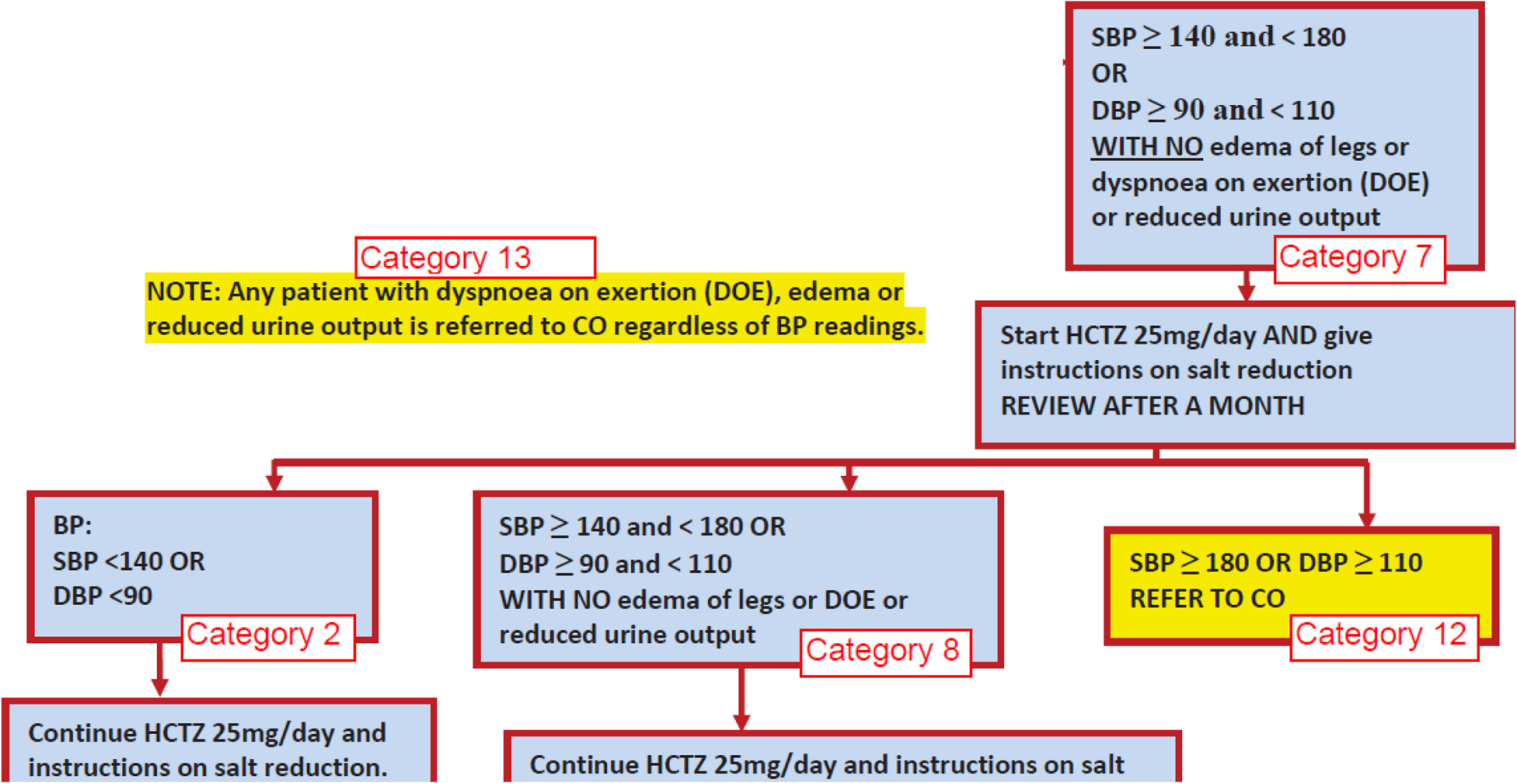
Encounter frequency and management plan by patient category. A portion of the AMPATH Hypertension clinical protocol is illustrated, demonstrating different patient categories, each associated with its respective encounter frequency and management plan. In this example, category 7 refers to a patient with elevated blood pressure not yet on medications, who enrolls in nurse-managed hypertension care and initiates pharmacotherapy. This patient can improve (category 2), remain unchanged (category 8), or worsen (category 12 or 13).

The AMPATH clinical protocol for hypertension management being used at the time of the model development and data collection (2013–2014) was derived from the evidence-based standards of the Seventh and Eight Reports of the Joint National Committee (JNC) on Prevention, Detection, Evaluation, and Treatment of High Blood Pressure (JNC 7 and JNC 8) [20, 21] using drugs contained in the Kenyan national formulary [22] and has been shown to reduce blood pressure among patients with hypertension [23]. This clinical protocol (Appendix) was used to determine “patient states” (e.g., systolic blood pressure (SBP) ≥ 140 mmHg and < 180 mmHg), each of which was linked to a specific management plan (e.g., “educate on lifestyle changes and repeat blood pressure in one month”). This yielded 26 unique “patient categories,” each of which combined a patient state with a management plan (Fig. 2). We defined three possible outcomes for each patient category: improvement (BP controlled), no clinical change, and worsening. Each of these outcomes would then determine the subsequent patient state (e.g., SBP remains ≥ 140 mmHg and < 180 mmHg upon repeat measurement) and linked management plan (e.g., “enroll into nurse-managed care and initiate pharmacotherapy”), corresponding to the subsequent patient category.

**Fig. 3:**
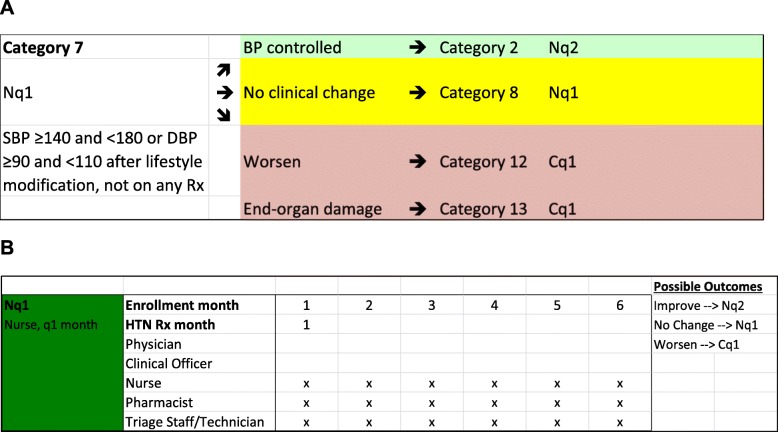
Change in frequency of required nurse visits (**a**) and schedule of encounters with providers (**b**) for an individual patient. **a** A patient being seen by a nurse every month (Nq1) can either improve and transition to being seen every 2 months (Nq2), remain unchanged and continue to be seen every month (Nq1) or worsen and require referral to a higher level of care (clinical officer) to be seen every month (Cq1). **b** The schedule of encounters for each patient, demonstrating that the Nq1 patient sees the nurse, pharmacist, and triage staff during each monthly trip to the health facility.

The 26 patient categories were collapsed into six “clinician-frequency combinations.” More clinically complex patients are seen by higher-level providers, with higher frequency. In the example above, a patient enrolled in nurse-managed care who has initiated pharmacotherapy would be seen by a nurse every month (Fig. 3a); in contrast, a patient with diabetes and hypertension (reflecting greater clinical complexity) would be seen by a clinical officer every month. For every clinician-frequency combination, we created a schedule of “encounters” (which clinician or staff person a patient sees during each patient “trip” to the health facility). In the example above, for the patient enrolled in nurse-managed care, the patient would encounter the nurse, the pharmacist (for medication refill), and the triage staff (for intake and registration) at every trip. At the end of a 6-month period, the patient category and clinician-frequency combination would be re-assessed, allowing for patients to either improve, remain in the same state, or worsen, with a corresponding change in category and clinician-frequency combination (Fig. 3b). We created a model for 3 years, with 6-month re-assessments based on usual clinical practice according to the AMPATH clinical protocol.

Similar to the workforce estimation model developed for HIV care in Mozambique [13], our model was programmed using spreadsheet technology in Microsoft Excel 2010 (Redmond, WA). Variables that can be modified include both demand (need) and supply (capacity) variables and include the following:

1. Demand (need):
  - Distribution of patients across patient categories and clinician-frequency combinations (i.e., what proportion of patients need to be seen by a nurse vs. a physician; what proportion of patients need to be seen every 1 month vs. every 6 months).
  - Total number of patients, either as target/expectation at the end of each year, or number enrolling per month.
  - Patient attrition due to loss to follow-up.
2. Supply (capacity):
  - Number of encounters per day per FTE for each type of provider (e.g., nurses can see more patients than physicians due to lower-complexity patients requiring less time per encounter).
  - Total number of days available for clinical encounters.

Three worksheets are required to generate the estimate of need/demand. Worksheet A has rows that correspond to every clinician-frequency combination linked to every potential outcome after 6 months. The columns correspond to each month in the 3-year period (36 months). Each cell corresponds to the number of encounters that a patient has with each type of provider at that particular time point.

Worksheet B has a similar structure to worksheet A with rows corresponding to every clinician-frequency combination linked to 6-month outcome, as well as 36 columns. However, each cell corresponds to the total number of encounters per type of provider, taking into account the inputs with respect to the total number of patients per month.

Worksheet C is from the health system perspective and also has 36 columns for the 3-year period. The number of rows consists of the number of provider types multiplied by 36 (number of months in the model).

A fourth worksheet, worksheet D, takes inputs for both need and capacity and generates the FTE requirement for each year, according to Eq. (c) above. The model allows for two different scenarios. One scenario allows the user to enter the total number of patients enrolled at the end of each year—this scenario allows for flexibility to ramp up enrollment in subsequent years after starting with relatively slower enrollment. A second scenario allows the user to enter the number of new patients enrolled per month—this scenario mimics more of a “steady state” of new patients being enrolled at a relatively constant rate when the clinical program is more established.

## Results

Results from the Delphi surveys indicated that as of 2014, an estimated range of 3000–5000 patients was being treated by an estimated range of 10–70 nurses. By the end of 2015, there was a projected increase of 2000–6000 more patients, with an additional 10–80 nurses required to treat the greater need.

Results from the time-motion studies identified that among the hypertensive patients, approximately 60% required follow-up in 1 month, 32% in 2 months, and 8% in 6 months. Results from our time-motion studies and FGDs estimated that for nurses, the available patient contact time was 720 min per month (assuming 2 days per month dedicated to seeing hypertension patients in clinic, and 6 h per clinical day available to see patients), with the time per clinical encounter equaling 8.9 min (range 3.5–20.2).

Acknowledging that the inputs can be varied, as outlined above, we offer examples illustrating the two scenarios described above. We assume an equal probability of changing categories over time, specific to each starting category, but that parameter can be modified as required to fit program-specific need data. In the first example, we start year 1 with zero patient volume and assume 1000 patients at the end of year 1, 3000 patients at the end of year 2, and 7000 patients at the end of year 3 (ramp-up scenario). Full staffing requirements, from the system perspective, are summarized in Table 1. In summary, 4.8 nurse FTE would be required in year 1, 13.5 nurse FTE in year 2, and 30.2 nurse FTE in year 3.

**Table 1:**
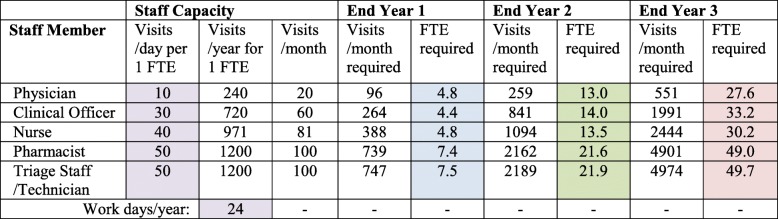
Workforce requirements at years 1, 2, and 3, according to the ramp-up scenario

Work days per year is based on an estimation that hypertension patients are seen on 2 days per month

In the second example, we start year 1 with zero patient volume and assume a constant monthly enrollment of 100 patients (steady-state scenario). Full staffing requirements, from the system perspective, are summarized in Table 2. In summary, 5.8 nurse FTE would be required in year 1, 10.5 nurse FTE in year 2, and 14.3 nurse FTE in year 3. Other staff categories for each time period are provided in Table 2.

**Table 2:**
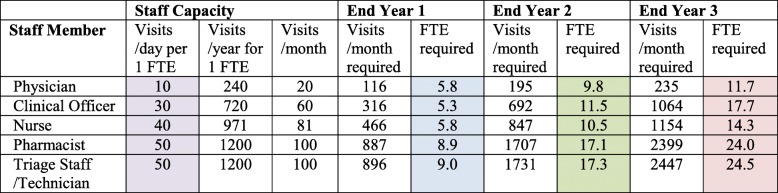
Workforce requirements at years 1, 2, and 3, according to the steady-state scenario

Work days per year is based on an estimation that hypertension patients are seen on 2 days per month

## Discussion

We present here a workforce estimation model for hypertension management that takes into account patient need and health system capacity, resulting in full-time equivalent health worker staffing estimates for hypertension management in rural western Kenya. We used a triangulation approach, combining data from key informant interviews, focus group discussions, a Delphi exercise, time-motion studies, and clinical management protocols, in order to generate inputs for the need and capacity parameters of the workforce estimation model. Based on the AMPATH hypertension treatment protocols, we were able to categorize patients according to clinical state, frequency of clinical encounters, and outcome from each encounter. The Excel-based model allowed for either ramp-up (increasing number of patients per year) or steady-state (constant enrollment per month) scenarios, thus yielding FTE requirements for nurses and other cadres of health worker at each yearly time point.

Our model allows for key inputs to be varied. For need, the model can accommodate variations in the number of patients, proportion of patients within each category, percentage of patients who change categories over time, and required number of clinical encounters per month. With respect to the capacity, the model can be modified according to the number of days per month when hypertension patients are seen in the health facility, number of hours available per health worker for clinical encounters, and amount of time spent per clinical encounter. While we presented two illustrative scenarios, the various inputs can be modified as required in order to be specific to each setting and program. A live Excel workbook is provided as an Additional file 1 for those interested to work directly with the model.

Our approach for generating the need and capacity inputs consisted of combining results from distinct participant groups and data sources, using a combination of qualitative methods, a Delphi exercise, quantitative time-motion studies, and categorization of established clinical protocols. This approach allowed for triangulation across participant characteristics, study procedures, deductive and inductive content coding, and analytic approaches. Triangulation is a process of comparing the results from multiple distinct analytic approaches or sources of data, in order to enhance construct validity and trustworthiness of inferences [24, 25]. While there were substantial consistency and congruence of our results, there were also some notable variation. These differences reveal the diversity of stakeholder perspectives. Such differences also highlight the importance of being able to vary the inputs in the model, in order to generate ranges of estimates useful for program planning and policy formulation.

A key component of the workforce estimation model was the translation of the AMPATH hypertension clinical protocol into a discrete number of “clinician-frequency” combinations, each with a schedule of encounters, with re-assessment of status at 6-month intervals, as described in detail above. We elected to create six categories, with follow-up periods that ranged from 1 to 6 months depending on the clinical provider and with no variability in the re-assessment time interval. While this might not mimic clinical practice exactly, we felt it was a reasonable approximation while still yielding a model that was manageable and feasible to manipulate. Depending on the complexity of the clinical algorithm, the granularity with which the end-user wishes to categorize different patient-clinician combinations, the frequency of re-assessments, and the likelihood of patients changing categories over time, this process can be modified.

Given the burden of hypertension and other non-communicable diseases (NCDs) around the world, it is critical that governments, health systems, and health facilities account for NCD-related health workforce requirements in their planning and implementation processes [26]. The World Health Organization (WHO) Global Strategy for human resources for health states that the supply of health care workers needs to reflect the increasing burden of NCDs [27], and this type of health workforce estimation model can assist health facilities, programs, and governments with human resource planning. While we have limited our model and analysis to health workers within the formal health system, other cadres of health worker, such as community health workers, can also positively impact outcomes for hypertension and other NCDs [28, 29]. Our model can be modified to take into account community health workers and other allied health professionals, as required.

One limitation of our approach is that, as with any model, the assumptions and inputs may not reflect exactly the reality on the ground. While we acknowledge that this is an issue, we feel that the model is flexible enough to accommodate variations in the inputs as required to match alternative scenarios. Second, we assumed that no patients were lost to follow-up or died. However, this parameter can be incorporated into the model as required, and workforce FTE estimations would be adjusted accordingly. Third, the needs-based approach proposed here assumes that hypertension-related health care should be provided according to need irrespective of other considerations. However, other factors may determine healthcare delivery, including costs, competing health priorities, cultural considerations, and societal values. Finally, we acknowledge that other models of health workforce estimation are available, such as task-based, demand-based, health-workforce-to-population ratio, service target-based, and probabilistic supply-demand matching, and each has its respective benefits and drawbacks [30–32]. Nevertheless, the current proposal is a critical first step in establishing a benchmark for health workforce estimation for hypertension management in LMICs.

## Conclusions

A needs-based workforce estimation model yielded full-time equivalent estimates for different health worker cadres required for hypertension management in western Kenya. The model is able to provide workforce projections that are useful for program planning, human resource allocation, and policy formulation. This approach can serve as a benchmark for future studies to manage chronic diseases in low-resource settings worldwide.

## Additional file

Additional file 1: Draft Excel model 20160508 BACKUP. (XLSX 677 kb)

## Data Availability

This study complies with the NIH Public Access Policy, which ensures that the public has access to the published results of NIH-funded research, and therefore, all results have been (and will be made) available from final peer-reviewed journal manuscripts (including this one) via the digital archive PubMed Central upon acceptance for publication. The data can be made available to other investigators upon formal request made to the Academic Model Providing Access to Healthcare (AMPATH) Research Manager.

## Abbreviations

AMPATH: The Academic Model Providing Access to Healthcare Partnership
BP: Blood pressure
CDM: Chronic disease management
DBP: Systolic blood pressure
FGD: Focus group discussions
FTE: Full-time equivalents
HIV: Human immunodeficiency virus
JNC 7: Seventh Report of the JNC
JNC 8: Eight Report of the JNC
JNC: Joint National Committee on Prevention, Detection, Evaluation, and Treatment of High Blood Pressure
KII: Key informant interviews
LMIC: Low- and middle-income countries
NCDs: Non-communicable diseases
SBP: Systolic blood pressure
WHO: The World Health Organization
